# Correction between the Morphology of Acromion and Acromial Angle in Chinese Population: A Study on 292 Scapulas

**DOI:** 10.1155/2018/3125715

**Published:** 2018-11-11

**Authors:** Xiaoguang Guo, Min Ou, Gang Yi, Bo Qin, Guoyou Wang, Shijie Fu, Lei Zhang

**Affiliations:** ^1^Department of Orthopedics, Affiliated Traditional Chinese Medicine Hospital of Southwest Medical University, Luzhou, 646000, China; ^2^Academician Workstation in Luzhou, Luzhou, 646000, China; ^3^National Key Discipline of Human Anatomy, School of Basic Medical Sciences, Southern Medical University, Guangzhou, 510000, China

## Abstract

*Introduction. *The acromion is a small section of the scapula which extends anteriorly from the spine of the scapula and the acromial angle (AA) is a prominent bony point at the junction of the lateral border of the acromion and the spine of the shoulder blade. As is well known, the morphology of the acromion and the acromial angle are important as their anatomical variation may contribute to shoulder pathologies. However, few people have studied the morphology and the association between the acromion and the acromial angle. The study explores the acromion and the acromial angle in the anatomical morphology and the association, providing an anatomical basis for clinical diagnosis and treatment.* Material and Methods.* A total of 292 dry, intact scapulae (152 right, 140 left) were used in the study. Three types of the acromion were already measured, type I(flat shape), type II (curved shape), and type III (hooked shape), respectively. Three types of the acromial angles were also measured in this study, C shape, L shape, and Double Angle shape.* Results. *The research result shows that C shape and L shape were the most common, while Double Angle shape was the least common. C shape was often related to type I (flat shape) and L shape was often related to type II (curved shape).* Conclusions. *The presented data provides precise and well-sorted information about the acromion and the acromial angle variation in Chinese population, contributing to diagnosis and treating in shoulder pathology.

## 1. Introduction

The acromion is a small section of the scapula which extends anteriorly from the spine of the scapula. Traditional method of classifying the acromion was by the shape of its undersurface [1], it can be classified as type I (flat shape), type II (curved shape), and type III (hooked shape) when viewed in the sagittal plane. The acromial angle (AA) is a prominent bony point at the lateral border of the acromion and the spine of scapula. It is often used as an acupoint location by anatomical landmark. Three types of the acromial angle were measured in our study, respectively, as C shape, L shape, and Double Angle shape.

In pathology, the morphology of the acromion was associated with a variety of disorders and contributes to pathologic conditions in the shoulder [2]. For example, a lateral extension of the acromion plays an important role in the aetiology of degenerative tears of the supraspinatus tendon [3]. And the morphology of the acromion in calcific tendinitis differs from controls without subacromial pathology [4]. In 1972, Neer CS [5] suggested that changes in the acromial morphology cause the subacromial impingement and impingement syndrome. Though the acromial morphological variation was related to the subacromial impingement, the causal relationship of them should be further explored [6]. The theory, for the impingement syndrome of the rotator cuff muscles, classified the causative agents as anatomical and functional. The anatomical causes included the morphology and inclination of the acromion [1]. Moreover, indications for acromioplasty were based on clinical symptoms were generally supported by changes in the acromial morphology.

Simultaneously, the morphology of the acromial angle was also important in treatment of shoulder pathology [7]. Shoulder injection procedures had powerful diagnostic and therapeutic effects for the care of patients with pathologic conditions of the shoulder-girdle region [8], while it often operated without image guidance. The guidance before was based on practice of many shoulder surgeons. Although ultrasound guidance may improve the accuracy of injection to the putative site of pathology in the shoulder, it was not clear that this improves its effectiveness because of the significant additional cost [9]. The morphology of the acromion and the acromial angle may be useful to guide injections more accurately for clinicians. Furthermore, the hypothesis that the acromial morphology of patients with degenerative supraspinatus tendon tears differs from patients with traumatic tears was confirmed. Shoulders with degenerative tears showed a narrower subacromial space and a larger lateral extension as well as a steeper angulation of the acromion than with traumatic tears [10]. It was meaningful for the shoulder arthroscope surgery, which procedure relieves pain by decompressing the tight space around the rotator tendon in the shoulder. It removed the bursa and trims back the acromion bone for pain-free motion; therefore the morphology of acromial angle could give surgeons some reference.

Moreover, the morphology of the acromial angle was useful to the acupuncture and massage. It was used to determine the points near the acupuncture shoulder and through stimulating acupoints to alleviates acute and chronic shoulder pain. The data of the study can provide some additional factors to consider when choosing an optimal shoulder implant for Chinese population and creating future designs that may better accommodate this population.

Therefore, the morphology of the acromion and the acromial angle were important as their anatomical variation might contribute to shoulder pathologies. Many studies [1, 11–15] had reported the morphologic characteristics of the acromion. The reason for this special interest was the association between these features of the acromial morphology and the subacromial impingement syndrome and rotator cuff tears (RCTs), which were frequently seen in orthopedic practice. In 1997, Chambler et al. [16] described bony spurs are localized to the anterior undersurface of the acromion in the area of the coracoacromial ligament insertion. Acromial enthesophytes were thought to be the consequence of ossific fibers of the coracoacromial ligament. Bhatia DN et al. [17] described a more lateral extension of the acromion measured by the acromion index in patients with a rotator cuff tear, while the acromial angle was originally described by Banas et al. [18] who found a correlation between acromial angle and rotator cuff tears in patients with rotator cuff disease.

However, few of them paid attention to the shapes of the acromial angle. In addition, there was still uncertainty about the relationships between the acromion and the acromial angle. Therefore it could not provide more accurate anatomical positioning for the treatment of shoulder diseases. And it will reduce the cure rate of clinical shoulder disease.

The study was designed to investigate the anatomical shapes of the acromion and the acromial angle, in order to establish possible correlations, which may have clinical significance for better understanding of the morphology of the acromion and the acromial angle, enhancing better management of shoulder pathology by improving the accuracy of fixing the points and the efficacy of the treatment,and improving clinical cure rate.

## 2. Material and Methods

### 2.1. Material

A total of 292 completely ossified, dry, intact scapulas were used for the study from the Department of Human Anatomy in university of Southwest Medical University (SWMCTCM2017-0701). Of the 292 bones, 152 belonged to the right side and 140 belonged to the left one. All the scapulas were from mature specimens with unknown of the genders and exact ages. And then, bones were excluded if degenerative changes or surgical destruction were found. The sliding vernier caliper (accurate to 0.01 mm) was used to measure the line and the thickness of the acromion and the acromial angle, recorded in millimetres. The angular measurements were made with Adobe Photoshop CS6.

### 2.2. Methods

*Acromion.* Three types of the acromion were classified according to Bigliani et al. [1].

*Acromial Angle.* Three types of the acromial angle were measured in this study, C shape, L shape, and Double Angle shape: C shaped acromion angle: the lateral border of the acromion was rounded, with a curved arc shape, resembling the C-shaped; L shaped acromion angle: the lateral border of the shoulder had a significant turning, forming a bony protuberance, resembling the L-shaped; Double angle shaped acromion angle: the lateral border of the shoulder had two significant turnings, forming two bony protuberances. The following measurements were obtained. The breadth of the acromion was measured from the anteroinferior point (point A) of the acromion to the corner of the superior acromion (point B) of the acromion. Acromion length was measured by drawing a line from the anteroinferior point of the acromion to the tip of the acromion (point C) of the acromion. And the projection length of scapular spine was also measured from the anteroinferior point of the acromion to the midpoint of the internal scapular spine (point D). The thickness of the anteroinferior point of the acromion, the superior acromion, and the tip of the acromion of the acromion were obtained. The angle of the acromial angle and the angle between the line from the tip of the acromion to the superior acromion and the horizontal line were included. Point A represents the anteroinferior point of the acromion, point B represents the corner of the superior acromion, point C represents the tip of the acromion, and point D represents the midpoint of the internal scapular spine. Since the Double Angle shaped AA has two corners, point A represents the upper corner and point A' represents the lower corner (Figures 1 and 2).

### 2.3. Statistical Analysis

Statistical analysis was performed by using SPSS 21.0 software (Chicago, IL, USA). All data were presented by means ± standard deviation ($\overset{-}{x}$ ±s). Multiple comparisons between groups were made by one-way analysis of variance (one-way ANOVA) when the variances were homogeneous; nonparametric tests (Tamhane's T2) were used when the variances were not equal; Chi-square test was used for classification data, and statistical significance was defined as* p* < 0.05.

## 3. Results

### 3.1. Acromion

In acromion, type I (flat shape) and type II (curved shape) are the most common types accounting for 47.26% and 49.66%. But type III (hooked shape) only represents 3.08% (Figure 3).

### 3.2. Acromial Angle

In the acromial angle, C shape and L shape are the most common acromial angle accounting for 47.26% and 47.95%, whereas Double Angle shape is rare (only 4.79% of all acromial angle types) (Figure 4). Significant difference was existed between the C shaped AA and L shaped AA concerning the acromion breadth, the acromion length, and the distance of scapular spine. Additionally, C shaped AA related to L shaped AA in the angle of acromial angle (Table 1).

### 3.3. Correction

Different types of the acromial angle related to the acromion are not completely the same (X^2^=0.005, P=0.005<0.05/3). C shaped AA and L shaped AA related to the acromion differ in distribution (X^2^=0.001, P=0.001<0.05/3).

C shaped AA related to type I (flat shape) 58.0% is the most common type in the present study, related to type III (hooked shape) 1.4% is the less common type. The percent of the L shaped AA related to type II (curved shape) 59.3% is the highest (Table 2).

## 4. Discussion

This study reveals that type I (flat shape) and type II (curved shape) are the most common types of the acromion in present study accounting for 47.26%, 49.66%. While type III (hooked shape) is a rare form only accounting for 3.08%. In the acromial angle, C shape and L shape are the most common acromial angle accounting for 47.26% and 47.95%, whereas Double Angle shape is only 4.79% of all acromial angle types. No previous studies appear to have classified the types of the acromial angle and corrected it with the morphological types of the acromion. One of the main purposes of our study was to determine whether an association exists between the acromion and the acromial angle. The study corresponded with previous studies in which similar measurements have been made. Our study showed a statistically significant association.

Lateral acromial angle was originally reported by Banas et al. [18]. The mean acromial angle of all scapulas in our study was 108.06°±11.30 and thus was larger than in Banas' study (1995). This might be caused by the different measurements, which Banas et al. use the radiographs and one line was drawn along the superior and inferior most lateral points of the glenoid and represented the glenoid surface. Another line was drawn parallel to the acromion undersurface. The angle between these two lines represents the acromial angle. However we measured the acromial angle in anterior view directly.

It should be noted that, in addition to the three classical types of the acromion, there is a fourth, in which the middle third of the undersurface of the acromion is convex [11]. We did not classify type IV separately but combined this particular type with type I (flat shape) [19].

Bigliani et al. (1986) have reported a classification of the acromion according to the shape of its undersurface. They recognized three shapes of the acromion: flat, curved, and hooked. Nicholson et al. (1996) reported the distribution of the acromial morphological types was type I, 32%, type II, 42%, and type III, 26%. We studied in 292 scapulas in relation to its classification that type I and type II were slightly higher, while type III is obviously lower, compared with those in the present study. This is due to differences in the classification method and in the type of specimens among the various authors; but it may also reflect the subjects' medical condition or ethnic groups [12, 14, 15, 20]. There is also disagreement in the literature about how the acromial types develop [21–24]. The acromion is an important role in shoulder pathology diagnosis, treating, like shoulder injection, or even in the surgery.

Acromial angle may be meaningful to orthopedic surgeons during presurgical planning. Acromioplasty, or resection of the undersurface of the anterior acromion, is a common procedure that usually alleviates impingement pain and is important in the treatment of rotator cuff teams [20]. It can be difficult during arthroscopy, however, to evaluate how much bone needs to be nested. This factor is important because excessive acromioplasty can bead to fracture or can weaken the deltoid muscle origin and insufficient acromioplasty can fail to relieve symptoms [25]. Several articles have stressed the importance of evaluating anterior acromial shape on the arch view during presurgical planning. The amount of bone that needs to be resected to produce a flat acromion can be demonstrated by extending the posterior line drawn during measurement of the acromial angle.

In summary, the acromial angle is an objective and fairly reproducible measure of anterior acromial shape. The angle is useful in identifying patients with a greater likelihood of having a rotator cuff tear and in distinguishing patients with primary impingement from those with instability.

Limitations and suggested future research: This study has some limitations. Firstly, the classification and measurements were both carried out on dry specimens by using a micrometer and caliper. More precise measurements could be included by analyzing a patient CT scan with possible 3D reconstruction models. Secondly, we measured a number of 292 specimens of unknown sex and age that were collected from one university, preventing a comparison between genders and age differences. Thirdly, as this is the first classification available on the AAs according to morphological features on a Chinese population, we were unable to check the reliability of the classification types with other ethnic groups. These problems remain to be solved in the future.

## 5. Conclusion

Variations in the size and shape of the acromion and the acromial angle, which were observed in this study, will be of great help for surgeons to understand the shoulder pathology better and to decide the proper size of the glenoid component for the shoulder arthroplasty. The present study classified and measured the acromion and the acromial angle morphology in a large number of Chinese specimens. C shape and L shape were the most common, while Double Angle shape was the least common. C shape was often related to type I (flat shape) and L shape was often related to type II (curved shape). The presented data provides precise and well-sorted information about the acromion and the acromial angle variation in Chinese population, contributing to understanding of the acromion and the acromial angle.

## Figures and Tables

**Figure 1:**
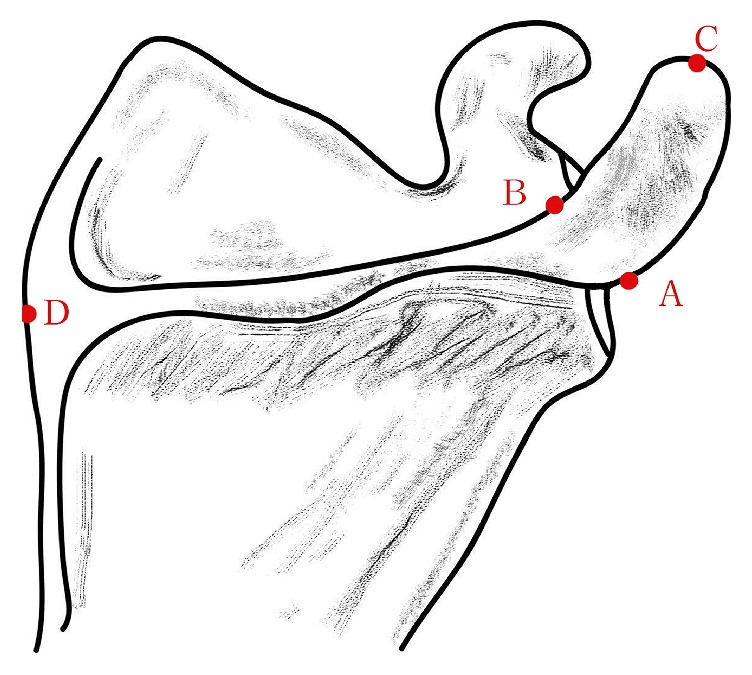
**A:** the anteroinferior point of the acromion (in the Double Angle shaped acromion angles, point A represents the upper corner and point A' represents the lower corner).** B:** the corner of the superior acromion.** C:** the tip of the acromion.** D:** the midpoint of the internal scapular spine.

**Figure 2:**
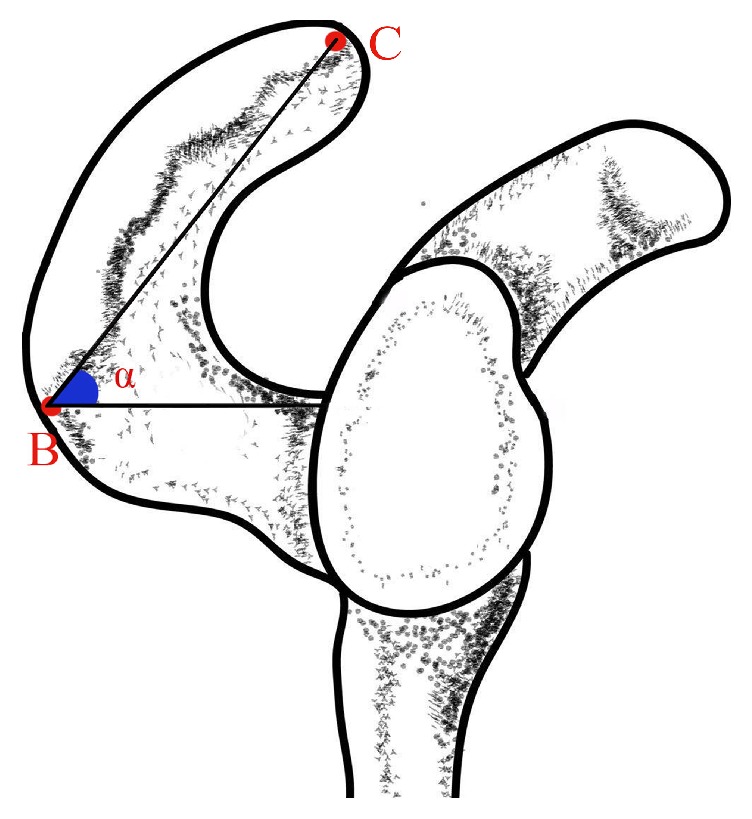
Measurement of the angle of inclination of the acromion** (**α**)**.

**Figure 3:**
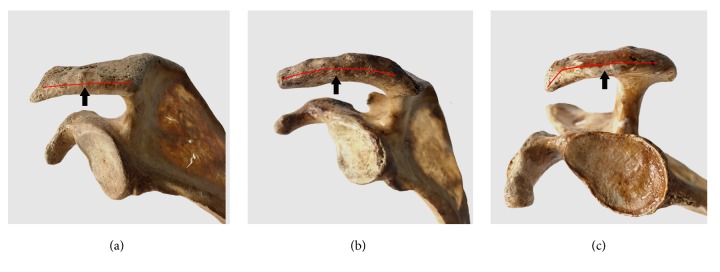
Three types of acromions according to Bigliani et al. (1986) (a) Flat: type I. (b) Curved: type II. (c) Hooked: type III.

**Figure 4:**
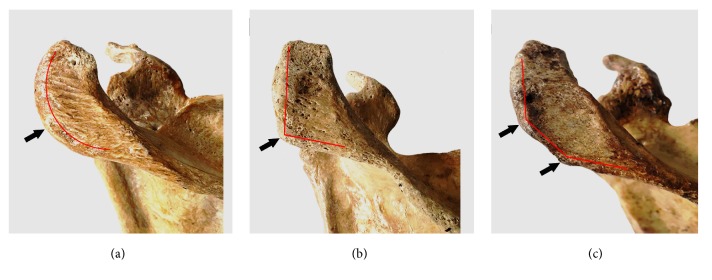
Three types of the acromion angles. (a) C shaped acromion angle: the lateral border of the acromion was rounded, with a curved arc shape, resembling the C-shaped. (b) L shaped acromion angle: the lateral border of the shoulder had a significant turning, forming a bony protuberance, resembling the L-shaped. (c) Double angle shaped acromion angle: the lateral border of the shoulder had two significant turnings, forming two bony protuberances.

**Table 1:** Measured values of the shape of the acromial angle based on classification.

	**N**	**AB (mm)**	**AC (mm)**	**AD (mm)**	**A (mm)**	**B (mm)**	**C (mm)**	**A(**°**)**	***α*(**°**)**
C shape	138	25.95±3.39*∗*^#^	40.94±5.50*∗*	111.10±10.19*∗*	6.83±1.92*∗*	6.06±1.64	5.70±1.37	110.22±11.66*∗*^#^	44.83±10.72
L shape	140	29.09±3.80	43.47±5.57	113.52±9.51	6.40±1.66	6.05±1.66	5.84±1.20	104.96±9.76^#^	45.71± 8.10
Double angle shape	14	28.80±4.57	43.60±5.37	110.76±8.45	7.07±1.92	6.29±2.14	5.74±1.58	117.69±11.88	48.64±6.30

A total of 292 dry, intact scapulas (152 right, 140 left) has been used for this table. AB, AC, AD, the comparison among three types in the acromion breadth, the acromion length, the distance of the scapular spine. A, B, C, the comparison among three types in the thickness of the anteroinferior point of the acromion, the corner of the superior point of the acromion and the tip of the acromion. A, ***α***, the comparison among three types in the angle of the acromial angle and the angle of inclination of the acromion.

*∗*P<0.05vs.L shape, #P<0.05vs.Double angle shape

**Table 2:** Overview of the association between the acromion and the acromial angle.

	**Type I (flat shape) (**%**)**	**Type II (curved shape) (**%**)**	**Type III (hooked shape) (**%**)**
C shape	58.0 (80)*∗*	40.6 (56)*∗*	1.4(2)*∗*
L shape	36.4 (51)	59.3 (83.00)	4.3 (6)
Double angle shape	50.0 (7)	42.9 (6)	7.1 (1)

A total of 292 dry, intact scapulas (152 right, 140 left) have been used for this table. Measuring the correlation between the acromion and the acromial angle.

*∗*P<0.05/3 vs. L shape.

## Data Availability

The initial data used to support the findings of this study are included within the supplementary information file.

## Supplementary Materials

Supplementary Materials This is the original data of the classification of acromion angle and the processing of SPSS software in this research, which includes the number of scapula and detailed measurement information.
